# The relationship between insulin resistance and recurrent pregnancy loss in assisted reproductive technology: A retrospective case-control study

**DOI:** 10.1097/MD.0000000000042373

**Published:** 2025-05-30

**Authors:** Yacong Cao, Miao Ding, Jingbo Chen, Chaofan Zhang, Fengyi He, Xiaojia Li, Sushi Jiang, Yanting Zou, Dongzi Yang, Xiaomiao Zhao

**Affiliations:** a Department of Obstetrics and Gynecology, Center for Reproductive Medicine, Sun Yat-sen Memorial Hospital, Sun Yat-sen University, Guangzhou, China; b Department of Reproductive Medicine, Guangdong Provincial People’s Hospital, Guangdong Academy of Medical Sciences, Southern Medical University, Guangzhou, China; c Department of Reproductive Immunology, Dongguan Maternal and Child Health Hospital, Dongguan, China; d Department of Clinical Nutrition, Sun Yat-sen Memorial Hospital, Sun Yat-sen University, Guangzhou, China; e Department of Reproductive Medicine Centre, Guangzhou First People’s Hospital, School of Medicine, South China University of Technology, Guangzhou, Guangdong China.

**Keywords:** assisted reproductive technology, insulin resistance, islet β-cell, recurrent pregnancy loss

## Abstract

This study aimed to investigate the association between insulin resistance (IR) and recurrent pregnancy loss (RPL) in patients undergoing assisted reproductive technology (ART). A retrospective analysis compared glucose and insulin indices (including fasting insulin [FINS], Homeostatic Model Assessment for Insulin Resistance [HOMA-IR], Homeostatic Model Assessment for Beta-Cell Function [HOMA-β], and area under the curve for insulin [AUCI] between RPL (n = 279) and non-RPL (n = 246) groups. Adjusted logistic regression models evaluated the correlation between IR-related indices and RPL risk. Compared with the non-RPL group, the RPL group exhibited significantly higher levels of FINS (10.67 vs. 8.57; *P* < .001), 1-hour insulin (1hINS) (110.86 vs. 74.75; *P* = .005), 2-hour insulin (2hINS) (89.47 vs. 67.94, *P* = .023), AUCI (117.08 vs. 114.16; *P* = .004), HOMA-IR (2.5 vs. 1.94; *P* < .001), HOMA-β (138.31 vs. 107.84; *P* < .001), the incidence of insulin resistance (63.47% vs. 47.03%; *P* < .001), and the incidence of HOMA-IR ≥ 2.14 (61.9% vs. 40.27%; *P* < .001). After adjusting for other factors, patients with IR had a higher risk of developing RPL compared with those without IR, with an odds ratio (OR) of 1.87 (95% CI: 1.18–2.94). Furthermore, an increase in FINS, HOMA-IR, and HOMA-β was associated with a significantly higher incidence of RPL, with OR values (95% CI) of 1.07 (1.03–1.12), 1.23 (1.03–1.48), and 1.01 (1.0–1.01), respectively. IR is an independent risk factor for RPL in ART patients, emphasizing the need for pretreatment interventions (e.g., lifestyle modifications or metformin) to improve insulin sensitivity and reduce miscarriage risk.

## 1. Introduction

Recurrent pregnancy loss (RPL), defined as the loss of 2 or more consecutive pregnancies before 24 weeks of gestation,^[1]^ is a challenging condition affecting couples attempting to conceive. It imposes significant emotional and physical burdens on couples striving to conceive. Surprisingly, up to 50% of RPL cases lack a clear underlying cause.^[2]^ However, certain factors such as genetic disorders, uterine pathologies, endocrine dysfunctions, autoimmune diseases, and environmental influences have been commonly associated with RPL.^[2]^ Recent studies have shed light on the potential involvement of insulin resistance, a metabolic condition characterized by impaired insulin action, in the development of this obstetric complication.^[3]^

Insulin resistance (IR) refers to a condition in which the target tissue fails to exhibit a normal coordinated response to normal levels of plasma insulin. This response includes the inhibition of endogenous glucose production, suppression of lipolysis, uptake of glucose from the bloodstream by cells, and net glycogen synthesis.^[4]^ It is commonly associated with conditions such as obesity, polycystic ovary syndrome (PCOS), and type 2 diabetes. IR affects multiple physiological processes, including glucose metabolism, lipid metabolism, and inflammation,^[5]^ which can have profound implications for reproductive health.

Research has indicated that patients with RPL exhibit a higher prevalence of IR compared with fertile controls, and IR is associated with increased miscarriage risk in PCOS patients undergoing assisted reproductive technology (ART).^[6]^ Furthermore, another study indicated that patients with IR and PCOS experience compromised ART outcomes, including pregnancy rate, implantation rate, and live birth rate.^[7]^ Moreover, the use of metformin, a medication that reduces IR, has shown promise in improving these ART outcomes for individuals with IR and PCOS.^[8]^

The intricate connection between IR and RPL calls for deeper investigation. This study aimed to evaluate whether IR is an independent risk factor for RPL in ART patients after adjusting for PCOS, obesity, and other confounders.

## 2. Methods

### 2.1. Study population

The study was retrospectively registered and conducted at Sun Yat-sen Memorial Hospital in Guangzhou, China, spanning from January 2012 to June 2018 (ethics batch number: SYSEC-KY-KS-2020-030). The participants enrolled in this case–control study had achieved pregnancy through ART, which encompassed methods such as in vitro fertilization (IVF), intracytoplasmic sperm injection, testicular epididymal sperm aspiration, percutaneous epididymal sperm aspiration, or frozen embryo replacement at the aforementioned hospital. All participants were women aged between 20 and 40 years old. The RPL group comprised women who had experienced 2 or more consecutive first-trimester subsequent abortions. On the other hand, the non-RPL group comprised women who underwent ART primarily due to male infertility or tubal-pelvic factors and had fewer than 2 first-trimester abortions.

**Figure 1.**
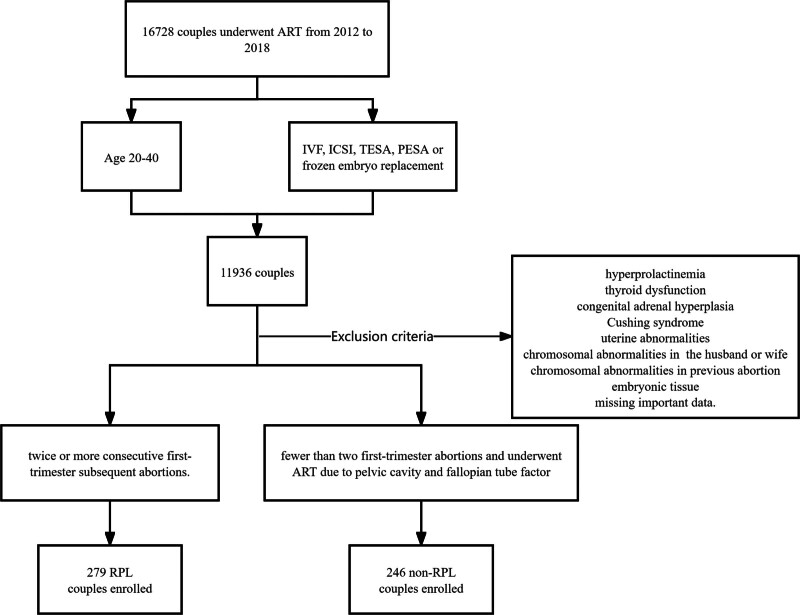
The patient screening flow chart.

The exclusion criteria for this study were as follows: cycles involving sperm or oocyte donation, cycles with preimplantation genetic testing, cases with obstetrical antiphospholipid syndrome, hyperprolactinemia, thyroid dysfunction, congenital adrenal hyperplasia, Cushing syndrome, or uterine abnormalities such as septate uterus, endometrial polyps, endometrial precancer or tumor, and moderate to severe uterine adhesions. Furthermore, cases where both the husband and wife had chromosomal abnormalities or cases with chromosomal abnormalities detected in embryonic tissues from previous miscarriages were also excluded from the study. The patient screening flow chart is shown in Figure 1.

Between 2016 and 2018, the principal investigator of the study selected a total of 246 control patients from the pool of outpatients. As our research group is concurrently conducting another study (ethics batch number: SYSEC-KY-KS-2021-094), we obtained relevant informed consent from the selected control group, and their offspring will be followed up as part of that study. Patients in the control group underwent screening for certain glucose metabolism parameters. During the screening process for the eligible control group, efforts were made to roughly match them with the case group in terms of age (20–40 years old), body mass index (BMI), living habits, educational background, and living environment. Any incomplete matches were rectified using appropriate statistical methods to ensure accurate and meaningful comparisons between the groups.

### 2.2. Anthropometric parameters

Height, weight, and waist and hip circumferences were measured while participants wore light clothing and were without shoes, ensuring a fasting state. Waist circumference was measured in a standing position at the midpoint between the lower ribs and the superior anterior iliac spine of the pelvis. Hip circumference was measured at the level of the pubic symphysis. To assess body composition, BMI was calculated using the formula: BMI = weight (kg)/height^2^ (m^2^); waist-to-hip ratio = waist circumference (cm)/hip circumference (cm).

### 2.3. Biochemical measurements and calculations

Blood samples were collected from the participants after a minimum of 8 hours of fasting. These samples were used to measure various parameters. The fasting plasma glucose (FPG) level and fasting plasma insulin (FINS) level were determined from the fasting blood samples. Additionally, blood samples were taken 1 hour and 2 hours after the ingestion of a 75 g glucose solution to measure plasma glucose levels at 1 hour and 2 hours postprandial glucose, as well as plasma insulin levels at 1 hour (1hINS) and 2 hours (2hINS).

To assess IR and β-cell function, the homeostasis model assessment of insulin resistance (HOMA-IR) and homeostasis model assessment of β-cell function (HOMA-β) were calculated. The area under the curve of glucose and the area under the curve of insulin (AUCI) were also calculated using the collected data. The specific formulas used to calculate these indices were as follows:


$$\begin{array}{l} \begin{array}{lll} & \begin{array}{l} HOMA\text{-}IR=FINS\left(U/mL\right)\times FPG\left(mmol/L\right)/22.5;HOMA\text{-}\beta \left(\%\right)=20\times FINS\left(U/mL\right)/\left[FPG\left(mmol/L\right)-3.5\right];AUCG=FPG/2+1hPG+2hPG/2; \end{array} & AUCI=FINS/2+1hINS+2hINS/2. \end{array} \end{array}$$

The accuracy of HOMA in the presence of hyperglycemia is limited; therefore, patients with prediabetes (impaired fasting blood glucose [IFG] or impaired glucose tolerance [IGT]) and diabetes are considered to have IR.^[9]^ The diagnosis of prediabetes and diabetes followed the guidelines set by the American Diabetes Association. IFG was defined as FPG ≥ 100 mg/dL (5.6 mmol/L), and IGT was defined as 2hPG ≥ 140 mg/dL (7.8 mmol/L). Diabetes mellitus (DM) was defined as FPG ≥ 126 mg/dL (7.0 mmol/L) and 2hPG ≥ 200 mg/dL (11.1 mmol/L). In the Chinese female population, there is still no conclusive cutoff value for HOMA-IR. A retrospective cross-sectional study in southeastern China reported a median and quartiles of HOMA-IR for females as 1.31 (0.93–1.88).^[10]^ Another national cross-sectional study suggested median and quartiles of HOMA-IR in Chinese women as 1.63 (1.17–2.29).^[11]^ Previous studies conducted in our reproductive center indicated a HOMA-IR cutoff value of 2.14 for diagnosing IR.^[12]^ Taking into account the results of these 3 studies, this current study adopted a HOMA-IR cutoff value of 2.14. In summary, IR was defined as meeting at least 1 of the following criteria: HOMA-IR ≥ 2.14, FPG ≥ 5.6 mmol/L, 2hPG ≥ 7.8 mmol/L, and/or 2hINS > 1hINS.

IR percentage = number of patients with IR/total number of patients in this group. Plasma insulin levels were quantified using the ACS180 SE autoanalyzer through chemiluminescence, following the manufacturer’s instructions (Bayer Diagnostics; Fernwald, Germany). Glucose levels were measured using the glucose oxidase assay, as per the manufacturer’s instructions, on the Beckman Coulter AU5800 automatic biochemical analyzer (America). These standardized procedures ensured accurate and reliable measurements of insulin and glucose levels for the study.

### 2.4. Statistical analysis

Statistical analysis was performed using R4.0.3 statistical software. Descriptive statistics were employed to assess the distribution of data and identify any outliers. Continuous variables with a normal distribution were presented as means ± standard deviations and compared using analysis of variance. For continuous variables with a non-normal distribution, medians (interquartile range, IQR) were reported and compared using the Kruskal–Wallis *H*-test, followed by post hoc Bonferroni correction. Categorical variables were expressed as relative frequencies and compared using the chi-square test.

Multifactor binary logistic regression analysis was conducted using the glm function. When the independent variable was categorical, the group with the minimum value served as the reference group. For continuous independent variables, they were directly included in the binary logistic regression model. Forest plots were generated using the forest package in the R language, presenting the results of the logistic regression analysis.

## 3. Results

### 3.1. Clinical characteristics of study subjects

From 2012 to 2018, a total of 16,728 patients underwent ART at our center. Among them, 525 patients in the case group met the selection criteria after excluding specific cases (shown in the flow chart, Fig. 1). These exclusion criteria ensured a more focused and reliable analysis of the remaining cases in our study.

A total of 525 subjects were included in the analysis, with 279 (53.14%) patients in the RPL group and 246 (46.8%) patients in the non-RPL group. Table 1 demonstrates that there were no significant differences in age, waist-to-hip ratio, and BMI between the 2 groups. However, notable distinctions were observed in other factors. The RPL group exhibited a shorter duration of infertility compared with the non-RPL group (2 vs 4; *P* < .001), with secondary infertility being the primary type in the RPL group (91.4% vs 36.59%; *P* < .001). Additionally, abnormal pelvic cavity and fallopian tubes were the main factors contributing to infertility in the RPL group (35.13% vs 21.14%; *P* < .001). None of the patients had a history of smoking, and there were no significant differences in exposure to secondhand smoking (husband smoking) between the 2 groups. The proportion of PCOS in the RPL group was significantly higher than that in the non-RPL group (18.28% vs 9.76%; *P* = .005). Moreover, the RPL group had a lower number of implantation failures compared with the non-RPL group (1 vs 1.5; *P* = .038).

**Table 1:** Clinical characteristics of recurrent loss of pregnancy (RPL) and nonrecurrent loss of pregnancy (non-RPL) women.

	Non-RPL (n = 246)	RPL (n = 279)	statistic	*P* value
Age (y)	34 (31,36)	34 (31,37)	−1.006	.314
WHR	0.81 (0.78,0.85)	0.82 (0.78,0.86)	−1.616	.106
BMI (kg/m^2^)	21.4 (20.01–23.12)	21.8 (20.2–24)	−1.903	.057
Duration of infertility (y)	4 (2–7)	2 (1–5)	6.256	<.001***
Infertility type			174.328	<.001***
Primary infertility, n (%)	156 (63.41%)	24 (8.6%)		
Secondary infertility, n (%)	90 (36.59%)	255 (91.4%)		
Infertility factors			166.939	<.001***
Pelvic cavity and fallopian tubes, n (%)	52 (21.14%)	98 (35.13%)		
Ovulation disorder, n (%)	6 (2.44%)	9 (3.23%)		
Endometriosis, n (%)	5 (2.03%)	2 (0.72%)		
Female multiple factors, n (%)	19 (7.72%)	90 (32.26%)		
Male infertility, n (%)	143 (58.13%)	22 (7.89%)		
Factors of husband and wife, n (%)	12 (4.88%)	35 (12.54%)		
Unknown reason, n (%)	9 (3.66%)	23 (8.24%)		
PCOS, n (%)	24 (9.76%)	51 (18.28%)	7.756	.005**
Husband smoking, n (%)	87 (35.37%)	104 (37.28%)	0.206	.65
Times of pregnancy loss (n)	0 (0–0)	2 (2–3)	−20.98	<.001***
Times of implantation failure (n)	1.5 (0–4)	1 (0–2)	2.077	.038*

Continuous values with an abnormal distribution were expressed as M (IQR) and were compared using Kruskal–Wallis H-test; categorical variables were expressed as relative frequencies and were compared using chi-square test.

BMI = body mass index, PCOS = polycystic ovary syndrome, RPL = recurrent pregnancy loss, WHR = waist-to-hip ratio.

* *P* < .05.

** *P* < .01.

*** *P* < .001.

### 3.2. Glucose metabolic characteristics of study subjects

Table 2 illustrates that there were no significant differences in FPG, 1-hour postprandial glucose, 2-hour postprandial glucose, and area under the curve of glucose between the RPL and non-RPL groups. However, several notable differences were observed between the 2 groups. The RPL group exhibited higher levels of FINS compared with the non-RPL group (10.67 vs 8.57; *P* < .001). Similarly, the levels of insulin at 1 hour (1hINS) (110.86 vs 74.75; *P* = .005), 2 hours (2hINS) (89.47 vs 67.94, *P* = .023), and the area under the curve of insulin (AUCI) (117.08 vs 114.16; *P* = .004) were significantly higher in the RPL group. Additionally, the RPL group had higher values for HOMA-IR (2.5 vs 1.94; *P* < .001) and HOMA-β (138.31 vs 107.84, *P* < .001) compared to the non-RPL group. The incidence of IR (63.47% vs 47.03%; *P* < .001) and the incidence of HOMA-IR ≥ 2.14 (61.9% vs 40.27%; *P* < .001) were significantly higher in the RPL group. However, there were no significant differences in the incidence of IFG, IGT, and peak insulin shift between the 2 groups.

**Table 2:** Glucose metabolic characteristics of recurrent loss of pregnancy (RPL) and nonrecurrent loss of pregnancy (non-RPL) women.

	Non-RPL (n = 246)	RPL (n = 279)	Statistic	*P* value
FPG (mmol/L)	5.1 (4.8–5.3)	5 (4.8–5.3)	0.98	.327
N	236	271		
1hPG (mmol/L)	8.59 ± 2.34	8.77 ± 2.27	−0.551	.582
N	71	144		
2hPG (mmol/L)	6.4 (5.55–7.68)	7.05 (5.73–8.3)	−1.594	.111
N	74	146		
AUCG	14.1 (11.95–16.58)	14.62 (12.92–16.61)	−0.868	.385
N	71	144		
FINS (mU/L)	8.57 (5.96–11.39)	10.67 (7.46–13.68)	−4.916	<.001***
N	231	257		
1hINS (mU/L)	74.75 (54.61–37.18)	110.86 (72.28–170.87)	−2.789	.005**
N	67	123		
2hINS (mU/L)	67.94 (40.44–113.59)	89.47 (51.91–157.13)	−2.278	.023*
N	68	124		
AUCI	114.16 (83.58–200.12)	177.08 (113.62–253.55)	−2.885	.004**
N	67	122		
HOMA-IR	1.94 (1.35–2.58)	2.5 (1.69–3.19)	−4.785	<.001***
N	226	252		
HOMA-β	107.84 (78.58–156.13)	138.31 (96.62–10.66)	−5.1	<.001***
N	226	252		
Insulin resistance (n(%))	111 (47.03%)	172 (63.47%)	13.816	<.001***
HOMA-IR ≥ 2.14 (n(%))	91 (40.27%)	156 (61.9%)	22.342	<.001***
FPG ≥ 5.6 mmol/L (n(%))	24 (10.17%)	27 (9.96%)	0.006	.939
2hPG ≥ 7.8 mmol/L (n(%))	18 (24.32%)	49 (33.56%)	1.978	.16
2hINS > 1hINS (n(%))	20 (29.85%)	49 (40.16%)	1.985	.159

Continuous variables with a normal distribution were expressed as means ± standard deviations and were compared using analysis of variance (ANOVA); Continuous values with an abnormal distribution were expressed as M (IQR) and were compared using Kruskal–Wallis H-test; categorical variables were expressed as relative frequencies and were compared using chi-squared test.

1hINS = 1-hour post-load insulin in OGTT, 1hPG = 1-hour post-load plasma glucose in oral glucose tolerance test (OGTT), 2hINS = 2-hour post-load insulin in OGTT, 2hPG = 2-hour post-load plasma glucose in OGTT, AUCG = glucose area under the curve during OGTT, AUCI = insulin area under the curve during OGTT, FINS = fasting insulin, FPG = fasting plasma glucose, HOMA-IR = homeostasis model assessment for insulin resistance, HOMA-β = homeostasis model assessment for β-cell function, RPL = recurrent pregnancy loss.

* *P* < .05.

** *P* < .01.

*** *P* < .001.

### 3.3. Risk factors related to glucose metabolism in recurrent pregnancy loss

**Figure 2.**
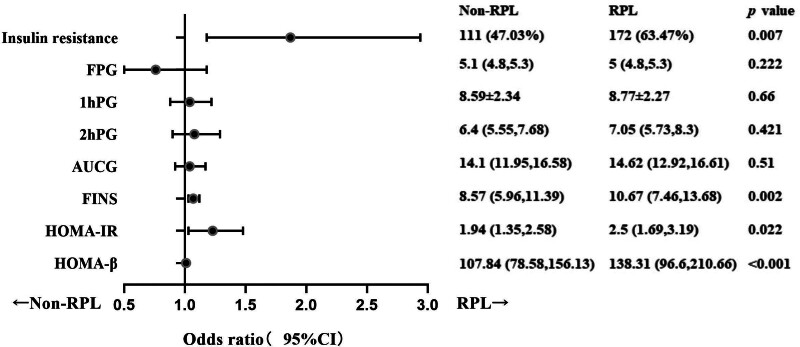
Forest plot for risk factors of glucose metabolism in RPL. 1hPG = 1-hour post-load plasma glucose in oral glucose tolerance test (OGTT), 2hPG = 2-hour post-load plasma glucose in OGTT, AUCG = glucose area under the curve during OGTT, CI = confidence interval, FINS = fasting insulin, FPG = fasting plasma glucose, HOMA-IR = homeostasis model assessment for insulin resistance, HOMA-β = homeostasis model assessment for β-cell function, OR = odds ratio, RPL = recurrent pregnancy loss.

Multivariate logistic regression analysis was employed to account for confounding factors such as years of infertility, infertility type, PCOS, and implantation failure times. The odds ratio (OR) values and 95% confidence intervals (CI) were calculated to assess the relationship between glucose metabolism-related indices and the risk of RPL. Table 3 displays the results of the analysis. After adjusting for other factors, it was found that patients with IR had a higher risk of developing RPL compared to those without IR, with an OR value (95% CI) of 1.87 (1.18–2.94). Furthermore, after adjusting for other factors, it was observed that an increase in FINS, HOMA-IR, and HOMA-β was significantly associated with a higher incidence of RPL, with OR values (95% CI) of 1.07 (1.03–1.12), 1.23 (1.03–1.48), and 1.01 (1.0–1.01), respectively. To visualize the data, a forest plot, as shown in Figure 2, was employed.

**Table 3:** The risk factors of glucose metabolism after adjusting other factors analysis for RPL population by logistic regression.

Glucose Metabolism Index	B	SE	z	*P* value	OR (95%C I)
Insulin resistance	0.625	0.232	2.69	.007**	1.87 (1.18,2.94)
FPG	−0.277	0.226	−1.221	.222	0.76 (0.49,1.18)
1hPG	0.036	0.082	0.439	.66	1.04 (0.88,1.22)
2hPG	0.075	0.094	0.805	.421	1.08 (0.9,1.29)
AUCG	0.04	0.06	0.659	.51	1.04 (0.92,1.17)
FINS	0.069	0.022	3.118	.002**	1.07 (1.03,1.12)
1hINS	0.001	0.002	0.535	.593	1 (1,1.01)
2hINS	0.003	0.003	1.049	.294	1 (1,1.01)
AUCI	0.001	0.002	0.816	.414	1 (1,1)
HOMA-IR	0.21	0.092	2.298	.022*	1.23 (1.03,1.48)
HOMA-β	0.007	0.002	4.125	<.001***	1.01 (1,1.01)

Multivariate Logistic regression was used to adjust the years of infertility, infertility type, PCOS, and implantation failure times, and the OR value and 95% CI of glucose metabolism-related indexes were calculated.

1hINS = 1-hour post-load insulin in OGTT, 1hPG = 1-hour post-load plasma glucose in oral glucose tolerance test (OGTT), 2hINS = 2-hour post-load insulin in OGTT, 2hPG = 2-hour post-load plasma glucose in OGTT, AUCG = glucose area under the curve during OGTT, AUCI = insulin area under the curve during OGTT, FINS = fasting insulin, FPG = fasting plasma glucose, HOMA-IR = homeostasis model assessment for insulin resistance, HOMA-β = homeostasis model assessment for β-cell function, RPL = recurrent pregnancy loss.

* *P* < .05.

** *P* < .01.

*** *P* < .001.

## 4. Discussion

This study conducted at Sun Yat-sen Memorial Hospital involved patients with RPL and controls undergoing ART. Various clinical and laboratory indices, including blood glucose metabolism, were evaluated in these patients. In comparison to the non-RPL group, the RPL group exhibited significantly higher levels of FINS, 1hINS, 2hINS, AUCI, HOMA-IR, HOMA-β, as well as a higher incidence of IR and HOMA-IR ≥ 2.14. After adjusting for other factors, it was observed that patients with IR had a higher risk of RPL than those without IR. Additionally, an increase in FINS, HOMA-IR, and HOMA-β was associated with a significantly higher incidence of RPL. These findings highlight the potential importance of IR in the development of RPL.

Weir and Bonner-Weir^[13]^ proposed a classification of diabetes progression consisting of 5 stages. The first stage, known as the insulin compensation stage, often involves IR. During this stage, there is an increase in the number and size of islet β-cells, resulting in increased insulin secretion while blood glucose levels remain normal. However, as the disease progresses, the mass and function of islet cells gradually decline, leading to decompensation in subsequent stages. HOMA-β is a parameter that provides an approximate assessment of islet β-cell function.^[14]^ In patients with RPL, although blood glucose levels were similar to those in the non-RPL group, elevated HOMA-β levels indicated a compensatory state of IR.

Several studies have investigated the relationship between IR and ART. In a prospective study involving nonobese, nonhyperandrogenemic patients with PCOS undergoing IVF, it was observed that serum insulin and HOMA-IR were significantly increased in PCOS patients compared to those with male infertility and unexplained infertility. However, there was no difference in the clinical pregnancy rate,^[15]^ suggesting that IR does not directly affect implantation. Another prospective observational study indicated that pregnancy rates decreased in patients with IR and PCOS after IVF. Oocyte development and embryo quality were not affected, suggesting that the impact of hyperinsulinemia on endometrial function and implantation may be the reason for the decreased pregnancy rate.^[7]^ Studies have shown that patients with RPL have a significantly higher prevalence of IR compared with fertile control groups.^[6,16]^ A meta-analysis involving 11,182 patients suggested that high BMI and IR were associated with an increased risk of spontaneous abortion in PCOS patients undergoing ART.^[17]^ This finding aligns with the conclusion of this study that IR is associated with an increased risk of miscarriage during ART.

Metformin, an insulin sensitizer, has been reported to reduce IR and miscarriage rates.^[18,19]^ However, a study by Diejomaoh et al^[20]^ demonstrated that IR was not significantly associated with the recurrent spontaneous abortion of unknown etiology. This discrepancy may be attributed to the limited number of studies available (35 in the recurrent miscarriage group and 30 in the control group). Nonetheless, our research findings suggest that IR may contribute to pregnancy loss. Fasting insulin levels in women who experienced recurrent miscarriages were significantly higher than those in the control group, although not to the extent of reaching IR.

However, our study did not specifically investigate the underlying mechanism of how IR leads to pregnancy loss. One crucial aspect of a successful pregnancy is the proper development of the endometrial decidual process, which plays a significant role in establishing a functional maternal-fetal interface. The decidualization process is essential for maintaining tissue homeostasis during the invasion of trophoblasts and provides protection against stress signals, including oxidative cell death.^[21]^ Abnormalities in endometrial decidualization can lead to impaired implantation and placentation, ultimately resulting in clinical conditions such as miscarriage.^[22]^ Research by Frolova et al^[23]^ has demonstrated that reduced glucose availability can hinder the expression of decidualization markers. Moreover, abnormal glucose metabolism has been shown to interfere with efficient decidualization both in laboratory settings and in living organisms. These findings suggest that disrupted glucose metabolism may contribute to incomplete embryo implantation and subsequent miscarriages.

A study conducted by Hu et al.^[24]^ on pregnant rats demonstrated that IR induced damage mediated by mitochondria as well as the subsequent imbalance of oxidative stress responses in the gravid uterus. Oocyte quality is another critical factor in female fertility, and research indicates that maternal metabolic changes can impact the follicular fluid microenvironment, leading to poor oocyte and embryo quality. In a separate study, elevated levels of insulin in follicular fluid were found to be associated with increased levels of C-reactive protein, indicating the presence of inflammation and heightened oxidative stress. These factors were linked to decreased developmental potential in the oocytes.^[3]^ Furthermore, Ou et al.^[25]^ conducted a study using an IR mouse model and found that IR contributed to oxidative stress and impaired mitochondrial function in mouse oocytes. These effects could disrupt the accurate transmission of mitochondrial DNA across generations, leading to poor oocyte quality and compromised embryonic development in IR mice.^[25,26]^

In addition, it is important to note that IR is closely associated with chronic inflammation throughout the body. IR triggers cellular stress, dysregulated responses, and stress responses. Moreover, cells targeted by insulin produce metabolites that further contribute to cellular stress generation both locally and systemically.^[27]^ Metabolic syndrome, characterized by IR, is considered an inflammatory disease, with vascular endothelial inflammation being a prominent feature. Prolonged exposure to excessive levels of free fatty acids and glucose can directly activate inflammatory pathways or increase the production of reactive oxygen species (ROS), thereby inducing inflammation.^[28]^ Research has suggested that children with chronic inflammatory diseases are often at a higher risk of developing IR. Chronic inflammation can lead to various abnormalities in the growth hormone/insulin-like growth factor 1 axis, further exacerbating IR.^[29]^ Additionally, increased influx of fatty acids and inflammatory molecules from other tissues, particularly visceral adipose tissue, triggers muscle inflammation, which negatively affects cardiomyocyte metabolism and contributes to IR.^[30]^ However, it is worth noting that adipose tissue inflammation plays a beneficial role in maintaining glucose metabolism balance. Research by Zhu et al.^[31]^ has shown that inhibiting inflammation in fat cells impairs adipose tissue function and promotes IR.^[31]^ Therefore, a multifaceted approach targeting various cellular stresses and stress responses could be explored to prevent or alleviate IR. Nonetheless, caution should be exercised when considering anti-inflammatory therapy in patients with RPL to alleviate IR. Anti-inflammatory drugs targeting vascular endothelium and skeletal muscle tissue hold promise as potential treatments for IR and may also serve as prepregnancy conditioning drugs for patients with RPL.

Given that this study is retrospective in nature, it is important to conduct a well-designed prospective study and further investigate the underlying mechanisms to better understand the role of IR in RPL. Additionally, investigating the underlying mechanisms involved in the relationship between IR and RPL is crucial. Further studies could explore the impact of IR on various aspects of reproductive processes, such as endometrial receptivity, decidualization, trophoblast invasion, and embryonic development. Molecular investigations, including gene expression profiling and signaling pathway analysis, may help elucidate the specific molecular pathways through which IR influences pregnancy outcomes. By integrating clinical observations with mechanistic studies, we can obtain a more comprehensive understanding of the complex interplay between IR and RPL.

## 5. Conclusions

In patients undergoing ART, IR is closely linked to RPL and may serve as one of the contributing factors. Patients with IR should be advised to improve insulin sensitivity through intervention before infertility treatment to reduce the risk of spontaneous abortion.

## Author contributions

**Conceptualization:** Yacong Cao, Dongzi Yang, Xiaomiao Zhao.

**Data curation:** Yacong Cao, Chaofan Zhang, Fengyi He.

**Formal analysis:** Yacong Cao, Fengyi He.

**Investigation:** Yacong Cao.

**Methodology:** Yacong Cao, Xiaojia Li, Sushi Jiang.

**Visualization:** Yacong Cao.

**Writing – original draft:** Yacong Cao, Miao Ding, Yanting Zou.

**Project administration:** Jingbo Chen.

**Writing – review & editing:** Jingbo Chen, Xiaomiao Zhao.

**Supervision:** Dongzi Yang, Xiaomiao Zhao.

**Funding acquisition:** Xiaomiao Zhao.
